# Deep Learning for CT Synthesis in Radiotherapy

**DOI:** 10.3390/bioengineering12121297

**Published:** 2025-11-25

**Authors:** Yike Guo, Yi Luo, Hamed Hooshangnejad, Rui Zhang, Xue Feng, Quan Chen, Wilfred Ngwa, Kai Ding

**Affiliations:** 1 Department of Biomedical Engineering, Johns Hopkins University, Baltimore, MD 21287, USA; 2 Department of Radiation Oncology and Molecular Radiation Sciences, Johns Hopkins University, Baltimore, MD 21287, USA; 3 Division of Computational Health Sciences, Department of Surgery, University of Minnesota, Minneapolis, MN 55455, USA; 4 Department of Biomedical Engineering, University of Virginia, Charlottesville, VA 22904, USA; 5 Department of Radiation Oncology, Mayo Clinic Arizona, Phoenix, AZ 85054, USA

**Keywords:** deep learning (DL), synthetic CT (sCT), radiotherapy

## Abstract

With the rapid development of artificial intelligence (AI), various deep learning (DL) methods have been introduced into radiation oncology. Among them, the generation of synthetic Computed Tomography (sCT) images has attracted increasing attention, as it supports different clinical scenarios, from image-guided adaptive radiotherapy (IGART) to the simulation-free workflow. This review provides a comprehensive overview of recent studies on DL-based sCT synthesis in radiotherapy from multiple imaging modalities, including Cone-Beam CT (CBCT), Magnetic Resonance Imaging (MRI), and diagnostic CT, and discusses their clinical applications in CBCT-based online adaptive radiotherapy, MRI-guided radiotherapy, and simulation-free workflows. We also examine the architectures of representative DL models such as convolutional neural networks (CNNs) and generative adversarial networks (GANs) and summarize emerging training strategies. Finally, we discuss current challenges of clinical translation of DL algorithms into clinical practice and suggest potential directions for future research. Overall, this paper highlights the potential of AI-driven sCT generation to advance treatment planning by reducing imaging burden, improving dose accuracy, and accelerating workflow efficiency, thus ultimately improving the treatment outcome of patient care.

## 1. Introduction

Medical image translation has emerged as a rapidly growing field within radiation oncology. Specifically, this denotes the transformation of images from one modality to another [1,2]. In the context of radiation therapy, Computed Tomography (CT) serves as the primary imaging modality, as it provides reliable electron density information essential for accurate dose calculation and treatment plan adaptation, i.e., replanning [3]. Nevertheless, CT imaging presents several limitations, including patient exposure to ionizing radiation and increased complexity and treatment delay in clinical workflows [4]. To mitigate these challenges, researchers have developed methodologies to generate synthetic CT (sCT) images from alternative modalities, such as Magnetic Resonance Imaging (MRI) and Cone-Beam CT (CBCT).

The advent of MRI-guided radiotherapy, facilitated by the development of MRI-Linac systems, has gained increasing attention [5,6,7,8]. These systems enable online adaptive treatment and provide real-time imaging throughout radiation delivery. A significant advantage of MRI lies in its superior soft tissue contrast, which enhances the precision of tumor localization and organ-at-risk (OAR) delineation [9,10]. However, as MRI inherently lacks electron density information critical to dose calculation, treatment planning and plan adaptation, conventional workflows often necessitate an additional planning CT scan. This requirement introduces several disadvantages, including potential image registration errors and additional patient exposure to radiation. MRI-only workflows have been proposed to address these issues by directly generating sCT images from MRI data. This innovation obviates the need for a separate CT scan, reduces registration uncertainty, and streamlines the clinical workflow [11,12,13].

Additionally, similar techniques have been applied to enhance the quality of CBCT, a 3D X-ray imaging technique commonly employed in image-guided adaptive radiotherapy (IGART) for both photon and proton modalities. However, CBCT images are affected by artifacts resulting from scatter noise and truncated projections, which restrict their utility for online plan adaptation [3,14]. Converting CBCT to sCT facilitates accurate dose computation and improves image quality.

Early sCT methods relied on deformable image registration or rule-based mapping. These approaches required careful tuning and were sensitive to variations in input. They also struggled with modality-specific artifacts [9]. Deep learning (DL) has changed this landscape. With the rise of convolutional neural networks (CNNs), generative adversarial networks (GANs), Transformers, and Diffusion models, sCT synthesis has become faster, more accurate, and less dependent on manual intervention.

Given the rapid development of this field, we aim to provide a comprehensive review of deep learning–based sCT generation. We begin by summarizing model architectures and training strategies and then explore clinical applications across different radiotherapy scenarios. Finally, we discuss current challenges and suggest future research directions.

Although several review papers have previously summarized developments in deep learning-based sCT generation [1,3,9,14,15,16]. While these works provide valuable overviews, they are often limited to either CBCT or MRI synthesis or focus primarily on technical architecture. In contrast, this review aims to provide a unified and up-to-date perspective across three major radiotherapy scenarios (CBCT-based online adaptive radiotherapy, MRI-guided radiotherapy, and simulation-free workflow), with an emphasis on model design, training strategies, and publicly available resources. Our goal is to bridge the gap between algorithm development and clinical translation, highlight open challenges and future directions for this rapidly evolving field, and provide a practical guideline for researchers seeking reproducible and open-source tools in this domain.

## 2. Public Dataset and Data Preprocessing

Before exploring specific model architectures, we first provide an overview of commonly used datasets and preprocessing techniques, which build the foundation for deep learning-based sCT synthesis.

### 2.1. Public Dataset

Publicly available datasets play a vital role in benchmarking sCT generation methods by enabling reproducibility, cross-study comparison, and model generalization across clinical scenarios. Below, we summarize several widely used datasets.

SynthRAD2023 dataset [17] provides paired MRI-CT and CBCT-CT images for brain and pelvic regions, collected from 540 patients in each anatomical site across three Dutch medical centers. All patients received external beam radiotherapy using photon or proton beam therapy. The extracted DICOM files were first converted to compressed NIFTI format and anonymized. To obtain uniform voxel spacing, images were resampled to 1 × 1 × 1 mm^3^ for the brain and 1 × 1 × 2.5 mm^3^ for the pelvis. Rigid registration between CT and MRI/CBCT was performed using Elastix [18] to address inter-modality misalignment. Binary masks of patient outlines were generated using thresholding and provided to standardize the field of view and support evaluation of synthetic CTs.

Building upon SynthRAD2023, SynthRAD2025 [19] significantly expands dataset scale and anatomical diversity. It includes 2362 cases, comprising 890 MRI–CT pairs and 1472 CBCT–CT pairs from five European university centers. The dataset spans head-and-neck, thoracic, and abdominal regions, with patients treated with external beam radiotherapy. Preprocessing followed the SynthRAD2023 protocol, with additional steps including defacing and deformable registration. However, deformable CTs are not provided for the training dataset to avoid biasing model development. Together, SynthRAD2023 and SynthRAD2025 datasets serve as large-scale, multi-institutional benchmarks for CBCT-to-CT and MRI-to-CT synthesis.

The Gold Atlas [20] contains MRI (T1- and T2-weighted) and CT images collected from 19 male patients across three Swedish radiotherapy departments. Patients with prostate or rectal cancer treated with curative radiotherapy were included. Nine pelvic structures were independently delineated by five experts, with consensus labels generated. An automated method (STAPLE [21]) was also used to produce probabilistic segmentation maps. This dataset is widely used for MRI-to-CT synthesis and downstream segmentation tasks.

Pelvic Reference Data [22] includes 58 pelvic CBCT-CT pairs with expert-annotated anatomical landmarks. These landmarks were used to derive reference rigid and affine transformations, which serve as ground truths for registration benchmarking. While primarily intended for registration, the dataset is also suitable for CBCT-to-CT synthesis studies.

Pancreatic-CT-CBCT-SEG dataset [23] comprises 40 CT and CBCT pairs from patients with locally advanced pancreatic cancer receiving ablative radiotherapy in deep-inspiration breath-hold mode. Each patient has one planning CT and two CBCT scans at the time of treatment. Rigid registration was applied to align CBCTs to CT, followed by voxel-wise resampling. Both raw and resampled CBCTs are included [24]. The dataset is notable for the artifacts from variability in the breath-hold levels present in CBCT, making it a challenging benchmark for sCT generation.

4D Lung dataset [25] consists of 4D fan-beam CT (FBCT) and 7 weekly 4D CBCT scans collected from 20 locally advanced non-small cell lung cancer patients receiving chemoradiotherapy. The 4D images consisted of a 3D image set for each of 10 respiratory phases. Target volumes and OARs were delineated by an expert radiation oncologist on all 4D-FBCT scans and selected 4D-CBCT phases [26], enabling studies in synthetic 4D.

All datasets reviewed in this study are publicly available, and we have included their official links and last access dates in the references to ensure transparency and reproducibility. While additional institutional datasets may exist in the literature, our focus is on open-source datasets that support community benchmarking and cross-study comparison.

### 2.2. Preprocessing Techniques

Preprocessing is important in generating high-quality sCT images by addressing limitations in data acquisition and enhancing the performance and stability of deep learning models. Common preprocessing techniques can be broadly categorized into spatial alignment, intensity standardization, and shape uniformity.

- Spatial alignment: Rigid and deformable registration are frequently applied to align multimodal images into common anatomical space. Rigid registration is often sufficient for rigid structures such as brain, while deformable registration is preferred in regions with higher anatomical variability such as pelvis. These methods mitigate inter-modality misalignment and ensure anatomical correspondence across modalities [14,15];
- Intensity standardization: To account for scanner-related intensity variations, intensity normalization is commonly employed at the population or patient level, either through linear scaling or z-score standardization using dataset-specific means and standard deviations. Intensity clipping can remove extreme outliers and suppress noise artifacts, improving data homogeneity [27]. Some studies also apply histogram matching to align intensity distributions across scans [15];
- Shape uniformity: Resampling is used to standardize voxel spacing across datasets, while resizing ensures a consistent input shape compatible with the model architecture. These operations are particularly important when combining multi-center or multi-modal data with heterogeneous acquisition protocols [27,28];
- Others: For MRI-based synthesis tasks, techniques such as N4 or N3 bias field correction are applied to reduce low-frequency intensity inhomogeneities and improve soft tissue contrast. In addition, cropping and geometry correction may be applied to remove unnecessary background or correct for distortions, particularly in MRI [15].

Collectively, these preprocessing steps enhance model robustness, improve generalizability across patient populations and imaging protocols, and enable more reliable benchmarking across studies. When combined with appropriate data augmentation strategies, preprocessing serves as a foundational component of effective sCT model development. Comprehensive preprocessing pipelines have been described in several studies [3,14,29] and the SynthRAD2023 and SynthRAD2025 datasets also provide publicly available code implementations to facilitate standardized preprocessing [17,19].

## 3. Deep Learning Models

To generate sCT images from different modalities, such as CBCT or MRI, various deep learning models have been explored. These models can be categorized into four main groups: CNNs, GANs, transformer-based architectures, and Diffusion models. Each modeling approach offers unique advantages in addressing the challenges of artifact removal and intensity fidelity, which are core requirements for the clinical use of sCT.

### 3.1. Convolutional Neural Networks (CNNs)

**Figure 1:**
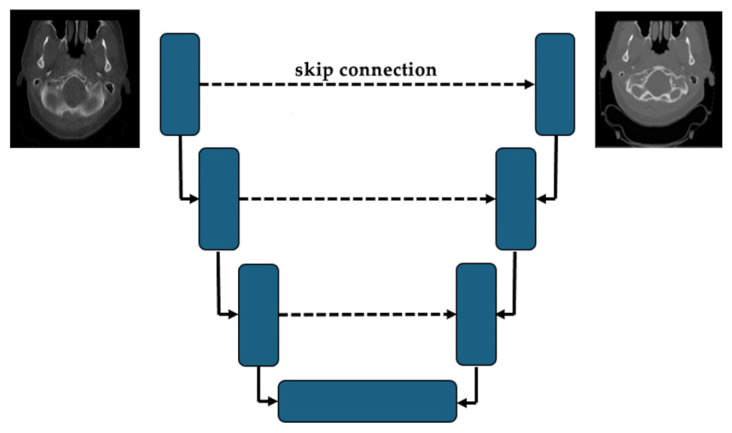
Overview of UNet-based CT synthesis from CBCT. The blue box stands for convolutional blocks, and the arrow represents the data flow from the encoder to the de-coder (flow of features).

CNNs have been applied in many sCT generation frameworks across both CBCT- and MRI-based modalities [30,31,32,33,34,35,36]. Among them, U-Net [37] and its variants have been the most widely adopted architectures. As depicted in Figure 1, due to the encoder–decoder structure and the inclusion of skip connections, U-Net models enable the preservation of spatial resolution and contextual information of the input modality throughout the network [38]. The nnU-Net (no-new U-Net) [39], a widely recognized framework for medical image segmentation, has also been adapted for MRI-to-CT synthesis [40]. To further enhance structural consistency, several modifications to the standard U-Net have been proposed. For instance, residual U-Net integrates residual blocks to mitigate vanishing gradients and facilitate deeper architectures [41]. Attention U-Net incorporates a self-attention scheme along the skip pathways to learn important features [42]. While CNNs are effective at learning local features, they may struggle to capture relationships between far-apart regions, which has led to interest in architectures such as transformers [14].

### 3.2. Generative Adversarial Networks (GANs)

**Figure 2:**
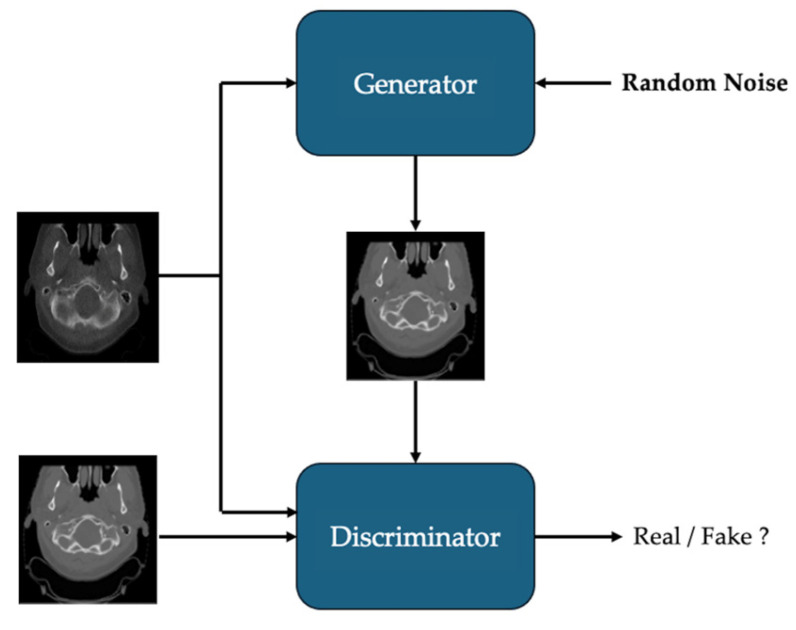
Overview of GAN-based CT synthesis from CBCT.

GANs have been explored for medical image synthesis due to their ability to generate perceptually realistic images [43,44,45,46,47]. A standard GAN consists of a generator and a discriminator trained in an adversarial framework, where the generator learns to synthesize images that mimic real data while the discriminator aims to distinguish between real and generated images [48], shown in Figure 2. This adversarial process promotes the generation of high-fidelity outputs [49]. In medical imaging, conditional GANs (cGANs) have become particularly common, enabling modality-to-modality translation by incorporating auxiliary information such as anatomical labels or imaging conditions [50,51,52]. A widely adopted variant, CycleGAN, introduces forward and backward mappings between domains, reinforced by a cycle-consistency loss enforced via two generators and two discriminators [53]. This mechanism alleviates the dependence on deformable registration and enhances robustness to anatomical mismatches [49].

To further improve image quality and anatomical preservation, attention mechanisms have been incorporated into GAN architectures. For example, attention-gated CycleGANs have been applied to correct motion artifacts in CBCT images [46], and attention-guided GANs have been proposed to emphasize clinically relevant features [54]. Multiple studies have benchmarked and extended GAN-based models for sCT generation. For instance, cGANs have been employed on multicenter pelvic datasets [55] while different generator architectures including DenseNet, U-Net, and EmbeddedNet were compared in [56], which demonstrated that ensemble learning achieved superior cross-domain generalization. Additional innovations include integrating histogram matching into CycleGAN for improved CBCT correction [57], spatial self-attention for structure-aware synthesis [58], and contrastive learning strategies tailored for 4D synthetic CT generation [59]. Despite these advances, GAN-based models often suffer from training instability, necessitating careful design and evaluation, especially in high-stakes applications like radiotherapy planning.

### 3.3. Transformer-Based Network

Transformer-based architectures, originally developed for natural language processing tasks, leverage self-attention mechanisms to capture long-range dependencies in sequential data [60]. Their ability to model complex structures has led to rapid adoption in vision tasks [61], including medical image synthesis. In the context of sCT generation, Transformer models have demonstrated strong potential in capturing global anatomical context and preserving structural integrity [62,63]. For instance, TransCBCT [62] employs a Transformer backbone for CBCT-to-sCT synthesis and was shown to outperform CycleGAN in both image quality and dosimetric accuracy. A residual visual Transformer that improves synthetic CT reconstruction by incorporating residual learning into the attention layers was introduced in [64]. To address region-specific synthesis challenges, a high-frequency information-guided synthesis model was proposed, which is a Transformer-based framework that effectively synthesizes multi-region pseudo-CTs from diverse MR sequences by emphasizing high-frequency anatomical features [65].

### 3.4. Diffusion Models

**Figure 3:**
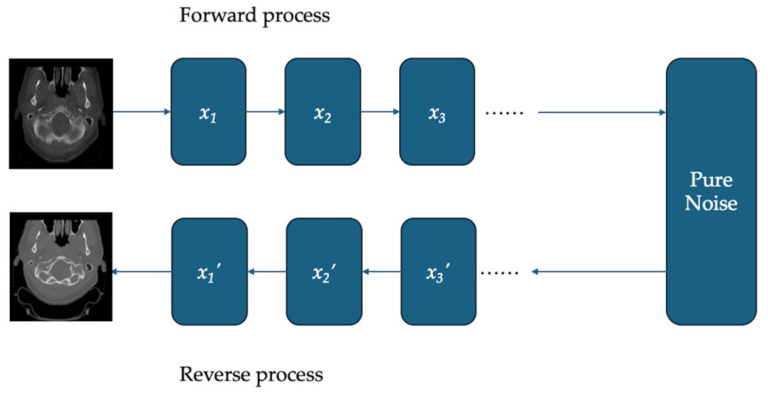
Overview of Diffusion-based CT synthesis from CBCT.

Another state-of-the-art network architecture for medical image synthesis is Diffusion models, which iteratively generate images by reversing a forward noise process, presented in Figure 3. These models transform random noise into structured data through a series of denoising steps and have shown strong generative capabilities in medical image translation tasks [2]. Diffusion models have been increasingly applied to improve image fidelity and anatomical consistency across modalities such as CBCT and MRI [66,67,68,69].

Several studies highlight the effectiveness of Diffusion models in surpassing traditional architectures like GANs. For example, it was demonstrated that a Diffusion-based CBCT-to-CT model outperformed GANs in lung imaging [66]. Additionally, an energy-guided Diffusion framework was proposed to enhance CBCT quality in unpaired settings, specifically tailored to meet the demands of adaptive radiotherapy [70]. To further enhance the flexibility and quality of synthesis, Diffusion Schrödinger bridge models were used to replace the standard Gaussian distribution with a learned prior distribution, improving both generation quality and efficiency [71]. On the MRI-to-CT front, a boundary-guided adversarial Diffusion model was designed to leverage unpaired data effectively [72].

### 3.5. Hybrid Models

To further exploit the strengths of multiple architectures, hybrid models have emerged as a promising direction in synthetic CT generation, combining the advantages of CNNs, transformers, adversarial training, and Diffusion mechanisms. These models aim to improve both global anatomical consistency and local structural fidelity. For instance, a generative-transformer adversarial-CNN framework integrates Transformer-based global modeling with adversarial and convolutional local refinements, achieving high-quality sCT synthesis even in low-dose CBCT scenarios [73]. Other hybrid designs have incorporated Transformer-Diffusion synergy. Hu et al. introduced a U-Net-based Diffusion model enhanced with Vision Transformer blocks to refine CBCT-to-CT translation [74]. Similarly, Swin-VNet (a Transformer variant) was used to guide MRI-to-CT Diffusion synthesis [75], while Viar-Hernandez et al. utilized SwinUNet in a Diffusion framework for CBCT-to-CT translation [76]. Another innovative example is the Global-Local Feature and Contrast learning (GLFC) framework by [77], which incorporates Mamba modules, designed for efficient long-sequence modeling, into a U-Net backbone to capture both global and local features. This method achieved state-of-the-art performance with improved Hounsfield Unit (HU) fidelity and structural similarity. In the GAN domain, Transformer modules have also been embedded into adversarial architectures. Hu et al. enhanced the CycleGAN model with Vision Transformer layers [78], while Rusanov et al. developed a Transformer-CycleGAN model that fuses cycle-consistency with attention-based global context understanding [79].

These hybrid approaches reflect a broader trend in medical image synthesis: rather than relying solely on a single model family, combining architectural paradigms can unlock new performance levels in terms of realism, structure preservation, and clinical applicability.

## 4. Training Strategies

To enable robust and generalizable sCT generation, various training strategies have been developed. These include various representations of input data, diverse supervision schemes, as well as learning paradigms.

### 4.1. Representation of Imaging Data

The dimension of input data in sCT model development is often categorized as 2D, 2.5D, or 3D, with each underlying a trade-off between computational efficiency and spatial contextual representation. Among them, 2D models have been the most widely employed due to their low computational requirements and ease of implementation, as they process each slice independently [2,15]. However, this absence of inter-slice context can lead to anatomical discontinuities across neighboring slices. In contrast, 3D models leverage volumetric inputs to learn spatial continuity and anatomical coherence across the full volume [75,76,80,81,82,83]. This also places considerable demands on computational resources and dataset size. To balance performance and effectiveness, 2.5D models have been introduced. These typically incorporate multiple consecutive slices as contextual input to 2D networks, thus enhancing spatial consistency without the full cost of 3D modeling [36,47]. In particular, Kondo et al. showed that incorporating neighboring slices into a 2D CNN improved spatial consistency in sCT generation [36].

Comparative evaluations of these dimensional strategies have reported mixed results. Neppl et al. observed a slight advantage of 2D models compared to 3D models, while both approaches achieved comparable dosimetric accuracy [84]. Findings from the SynthRAD2023 challenge further emphasize that optimal dimensionality may be dependent on imaging modality. Specifically, 2D models outperformed both 2.5D and patch-based 3D architectures for MRI-to-CT synthesis, while 3D models exhibited superior results in CBCT-to-CT synthesis across both pelvic and brain datasets [27]. These results contrast with earlier studies reporting that 2.5D or 3D models can outperform 2D counterparts in MRI-to-CT tasks [85,86,87]. This suggests that the effectiveness of dimensionality strategies may vary by imaging modality and anatomical site.

Anatomical view design has emerged as another important approach to improve structural consistency in sCT synthesis. Instead of depending exclusively on input dimensionality, several works have proposed training separate models for different anatomical planes (axial, coronal, and sagittal) and averaging their predictions to improve robustness. Spadea et al. pioneered this approach by averaging predictions from three independent CNNs [86]. Saint-Esteven et al. extended this idea using residual vision transformers [64]. Further, Yoganathan et al. trained a single-view axial model as well as a multiplanar model, which adopted a similar network as [86] and reported no statistically significant difference between these two models in dose prediction accuracy [42].

### 4.2. Supervision Paradigms

Most of the deep learning models are developed using pixelwise supervision and trained on aligned image pairs (MRI-CT or CBCT-CT). Pair training facilitates stable optimization with intensity-based losses, leading to superior HU accuracy and anatomical conservation [14,88]. Nevertheless, acquiring high-quality paired datasets presents several challenges. Both modalities are acquired with a significant time gap and repetitive usage of ionizing radiation, which is not acceptable in a vulnerable population like pregnant women. In addition, variations in the timing of acquisition might cause spatial discrepancy due to tumor progression. While deformable image registration can perform image warping, it can also introduce additional anatomical distortion or artifacts [89,90,91].

To address these limitations, unpaired training methods have been attracting more attention, as they could avoid complicated data preprocessing and are more adaptable to different kinds of institutions. CycleGANs are a popular choice since they are capable to learn the mapping between two modalities by imposing cycle-consistency constraints [43,44,92]. However, unpaired learning may suffer from model performance, such as compromised HU accuracy and poor anatomical structure preservation. To address these shortcomings, recent methods have attempted to incorporate anatomical priors into unpaired learning frameworks. For instance, a path- and bone-contour regularized training strategy was proposed, which can learn domain mappings in a shared latent space using neural Ordinary Differential Equations (ODEs) [91]. Likewise, Gong et al. developed a boundary information-guided adversarial Diffusion model, outperforming standard CycleGANs on pelvic MRI datasets [72]. These approaches improve structural accuracy in the absence of voxel-level supervision and have shown competitive results compared to traditional paired setups.

Depending on the paired datasets, most sCT methods adopt supervised learning to train the models. Due to the limitation of aligned datasets, unsupervised learning has been introduced to generate CT without ground truth labels. Early approaches utilized adversarial networks with additional structural constraints, such as CycleGAN proposed by [93], which outperformed traditional CycleGAN. Building on this, recent works leverage the flexibility of Diffusion models and representation learning. For example, Peng et al. developed a patient-specific Diffusion model utilizing a score-based method, generating sCT with reduced artifacts and high HU values [94]. Similarly, Zhang et al. introduced a disentanglement learning framework that shares the information of image pairs in the latent space [95]. Other innovations focus on edge and structural fidelity. Zhu et al. proposed an edge-aware unsupervised GAN to enhance boundary delineation and recover missing anatomical structures [96]. Moreover, Szmul et al. introduced an unsupervised framework capable of simultaneously synthesizing sCT and segmenting OARs, without relying on CBCT segmentations during either training or inference [97].

### 4.3. Learning Paradigms

Loss functions are essential to optimizing deep learning models, and many have been used in sCT synthesis. Traditionally, L1 and L2 losses, which are the average of the absolute difference and the squared error between the predicted and ground truth values, respectively, are often utilized in U-Net-, Transformer-, and Diffusion-based models to guarantee per-pixel correctness. Another commonly used metric is the structural similarity index (SSIM) loss that enforces structural consistency. Several studies include L1, L2 and SSIM losses in weighted sums with hyperparameters that are tunable to balance between pixel-level accuracy and perceptual similarity [1,14,98]. In GAN-based models, adversarial loss is the main factor for realistic improvement, which trains the generator to output images that are indistinguishable from real ones. Hybrid loss functions are proposed to further enhance the image quality [14,77,92,93,99,100,101]. These typically use pixel-wise losses (L1/L2) with region-of-interest (ROI)-oriented losses [77,99,100] or include perceptual losses based on pre-trained networks (e.g., VGG) to promote high-level structural and textural fidelity [92].

Another popular method for training is guided training, which is usually guided by either image frequency characteristics or anatomical structure. Frequency-guided learning has been widely used in recent works [65,68,102,103,104,105] which mostly employed Diffusion architectures. For example, Li et al. proposed a frequency-aware Diffusion model, which designs low- and high-pass filters to reconstruct information of intermediate frequency image content [68]. Building on this, Zhang et al. incorporated a high-frequency optimization module based on wavelet transform to improve textural details [103], while Luo et al. proposed a high-frequency smoothness constraint to maintain edge sharpness and fine structures [105]. In contrast, anatomy-guided models are also proposed to maintain the structural fidelity during the synthesis stage [72,93,99,106,107,108,109]. Yang et al. designed a structure-constrained GAN for unsupervised MRI-to-CT translation which incorporated a structure-consistency loss [93]. A dual-stream structure-aware GAN was proposed in [106] to capture the structural information within the output images. Additionally, bone region fidelity was highlighted by leveraging a multi-task network focused on bone density restoration by Kaushik et al. [99]. Transformer-based models have also introduced structural attention mechanisms to enhance the anatomical realism [109], and Yu et al. developed a multi-level, hierarchical discriminator to enhance the fidelity of synthesized MR images [108].

To mitigate data privacy concerns and promote multicenter research, federated learning has been integrated in the context of synthetic CT generation [58,110,111]. One of the earliest works is proposed by [110], which utilized a cross-silo horizontal FL framework for MRI-to-CT synthesis, allowing several institutions to collaboratively train the U-Net-based architecture without sharing any raw data. Based on this model, researchers further developed the method for CBCT-to-CT synthesis, focusing on privacy protection and multi-institutional collaboration with only decentralized training (i.e., without sharing raw patient data or creating different site-specific models) [111]. Although the FL framework shows great potential, its performance is constrained by limited data diversity, with the most prominent errors occurring in anatomical regions that are underrepresented in the training dataset.

The choice of training strategy plays a pivotal role in determining the robustness, generalizability, and clinical utility of sCT models. Depending on the anatomical region, imaging modality, and data availability, different training paradigms can significantly impact performance in downstream applications. In the next section, we review how these models are deployed across various clinical scenarios.

## 5. Application in Radiotherapy

CT synthesis serves as a cornerstone for enabling IGART. Depending on the imaging modality and clinical setting, sCT generation facilitates accurate dose calculations, motion management, and online adaptation, thereby enhancing both treatment precision and workflow efficiency. In this section, we categorize recent advances into three application domains: CBCT-based online adaptive radiotherapy, MRI-guided radiotherapy, and simulation-free workflow. To facilitate reproducibility, we summarize in Table 1 a selection of deep learning models for sCT generation that are publicly available. Only models with accessible code or pretrained weights were included.

### 5.1. CBCT-Based Online Adaptive Radiotherapy

The CBCT-guided ART offers daily treatment adaptation using pre-treatment imaging, which increases the target conformality and the OAR sparing [14,49]. However, the use of CBCT is restricted in the clinic due to inherent limitations, such as image artifacts, low soft tissue contrast and imprecise HU, which can be critical in dose-sensitive environments, such as those used proton therapy [34,112,113]. To solve these problems, deep learning–based CBCT-to-CT translation is a promising approach to provide dose-computable images from the CBCT without acquiring an additional CT and the additional radiation exposure [114,115,116].

An increasing number of works have investigated sCT generation in other body sites such as brain [74,117], head and neck [50,89,118], thorax [35,59,66], breast [114], pelvis [47,119], spine [120], nasopharynx [32,52].Among them, the thorax suffers from specific challenges related to respiratory motion, which has led to the development of 4D CBCT-to-sCT translation methods [59,121]. In addition, investigators have developed other methods such as low-dose CBCT-based sCT generation [116,122], and dual-energy CBCT-based synthesis [76,123,124] to improve soft-tissue representation and HU uniformity.

One of the most important goals is to reduce artifacts in CBCT-based imaging synthesis. Image degradation arising from scatter, cone-beam geometry, and motion can be addressed through deep learning approaches in either the projection or image domain [125,126]. Dual-stage networks that explicitly decouple artifact removal from sCT generation have also proven effective, particularly in spine and abdominal targets [120,127]. Cycle-consistent adversarial networks such as CycleGAN [112,114] and cGANs [52] facilitate domain translation without paired CT-CBCT data. In particular, hybrid pipelines, such as ARTInp [128] and GLFC [77] incorporate additional domain knowledge or attention mechanisms to remove noise and improve context-aware generation.

The accuracy of sCT images for dose calculation has been verified in quantitative research. Several studies report that dose-volume histogram (DVH) parameters calculated from sCTs match well with those of a reference CT to support clinical application, supporting their clinical feasibility [113,129]. Additionally, previous retrospective investigations have also demonstrated the ability of sCTs to trigger plan adaptation in proton therapy workflows, which can potentially lead to fewer repeat CTs being performed [129].

### 5.2. MRI-Guided Radiotherapy

MRI-guided radiotherapy provides superior soft tissue contrast without exposing patients to ionizing irradiation, thus making it a compelling imaging modality for treatment planning. However, its lack of direct association with electron density or HU prevents its application in accurate dose calculations [12,43].To address this issue, deep learning-based MRI-to-CT synthesis methods have been developed to produce sCTs from MRI [30,31]. These methods have been investigated in various anatomical sites, such as the brain [1,36,84], head-and-neck [33,64], pelvis [55,80,130], and thorax areas [51].

There is a growing interest in low-field MRI-based sCT due to its affordability and seamless integration with hybrid systems such as MRI-Linacs. While low-field MRI has an inherently lower signal-to-noise ratio than high-field scanners, low-field MRI provides a realistic alternative for real-time on-board imaging in IGART workflows. Recent studies have demonstrated the possibility of generating high-quality sCTs from low-field MR inputs using conditional GANs and residual transformer architectures [45,51,64]. These models have guaranteed anatomical accuracy as well as clinically acceptable dosimetric performance, which establishes low-field MRI as an alternate base for MRI-only planning pipelines.

Another challenge in MRI-to-CT synthesis is the heterogeneity of MR sequences used in clinical protocols. Commonly employed sequences (e.g., T1-weighted, T2-weighted, Dixon-imaging) differ considerably in tissue contrast, spatial resolution, and acquisition parameters. To address this, several studies have proposed various methods that generated sCTs from different MR sequences in a single model. For instance, Zimmermann et al. developed a sCT generator independent of MRI sequence utilizing 3D UNet architecture, trained on T1, T2, and contrast-enhanced T1-weighted images [82]. Zhong et al. designed MTT-Net that utilized multi-scale tokens-aware Transformer network suitable for various anatomical regions [131], while Zhao et al. proposed a high-frequency-information guided network to generate sCTs from different MR sequences [65].

As MRI-to-CT-based models are approaching clinical translation, the generalizability of these models across institutions, scanners and patient populations becomes more important. However, the collection of such large, varied datasets is typically made difficult by privacy laws and data-sharing policies. In response to this, recent research investigated federated learning for training the models jointly, without sharing raw patient data. For example, RadiaSync [58] showed that decentralized training over multiple sites can match the performance of centralized models. Also, at the multicenter level, models based on sCT have demonstrated to maintain high dosimetric accuracy and fidelity even when they are trained on heterogeneous datasets crowded by different sources of acquiring information [130,132,133].

### 5.3. Simulation-Free Workflow

The clinical motivation for simulation-free workflows especially lies in fast-progressing diseases such as non-small cell lung cancer (NSCLC), where radiotherapy plays an increasingly pivotal role in local tumor control and survival outcomes and the current delay in time-to-treatment initiation (TTI) in lung cancer radiotherapy has significantly compromised patient outcomes [134,135,136,137,138]. Studies have shown that each four-week delay in radiotherapy is associated with a 6–8% increase in mortality [139] and a 13% chance of disease upstaging due to new lymph node involvement or metastasis [140]. Despite these risks, conventional workflows often involve over four weeks of delay between diagnosis and treatment initiation due to sequential simulation CT acquisition, manual contouring, and plan generation [141,142,143]. Simulation-free strategies aim to bypass these bottlenecks by synthesizing planning-quality CTs from existing diagnostic imaging, thereby enabling faster and more streamlined treatment planning. In time-critical and resource-constrained settings, such workflows may significantly eliminate imaging redundancy, reduce radiation exposure, and improve patient access to timely care [144,145].

Recent deep learning techniques have expanded the territory of simulation-free workflow by predicting deformation vector fields (DVFs) that map diagnostic images into the planning geometry. Instead of generating sCTs directly, these models can produce anatomically aligned transformations that can map diagnostic CTs (dCTs) to dose-calibrated pCT-like representations [146,147,148]. For example, deepPERFECT learns DVFs from diagnostic-to-planning CT pairs to produce anatomically corrected sCTs for dose calculation [146]. Based on this, DAART further develops the concept to a full adaptive radiotherapy model, allowing for reducing the current median 4 weeks workflow to 2 weeks from diagnosis to treatment initiation [147]. Similarly, Zhu et al. applied a DVF prediction model to lattice radiotherapy showing high gamma pass rates and dosimetric consistency in vitro in complex abdominal plans [148].

Despite promising results, simulation-free workflows need to be able to account for anatomical differences, changes in patient positioning, and motion induced artifacts, especially in abdominal and thoracic regions. In addition, proper HU calibration is still of critical importance for proton therapy, as well as for highly conformal photon plans.

**Table 1:** Publicly available deep learning models for synthetic CT generation in radiotherapy.

Application	Paper	Dataset Publicity	Model	Code Link
CBCT to CT	[83]	Pancreatic-CT-CBCT-SEG; SynthRAD2023	3D Unet	https://github.com/MaxTschuchnig/EnhancingSyntheticCTfromCBCTviaMultimodalFusionandEnd-To-EndRegistration (accessed on 20 October 2025)
	[149]	private	CycleGAN, StarGAN	https://github.com/Paritt/sCT-via-StarGAN-and-CycleGAN (accessed on 20 October 2025)
	[77]	SynthRAD2023	Mamba-enhanced UNet	https://github.com/HiLab-git/GLFC (accessed on 20 October 2025)
	[150]	private	Physics-based network	https://github.com/Pangyk/SinoSynth (accessed on 20 October 2025)
	[67]	private	Diffusion	https://github.com/junbopeng/conditional_DDPM * (accessed on 20 October 2025)
	[68]	private; Organs at Risk dataset [151,152]	Diffusion	https://github.com/Kent0n-Li/FGDM (accessed on 20 October 2025)
MRI to CT	[91]	Gold Atlas; SynthRAD2023	Neural ODE-based	https://github.com/kennysyp/PaBoT (accessed on 20 October 2025)
	[40]	SynthRAD2023	nnU-Net	https://github.com/Phyrise/nnUNet_translation (accessed on 20 October 2025)
	[149]	private	CycleGAN, StarGAN	https://github.com/Paritt/sCT-via-StarGAN-and-CycleGAN (accessed on 20 October 2025)
	[131]	private	Transformer	https://github.com/SMU-MedicalVision/MTT-Net (accessed on 20 October 2025)
	[75]	private	Diffusion	https://github.com/shaoyanpan/Synthetic-CT-generation-from-MRI-using-3D-transformer-based-denoising-diffusion-model * (accessed on 20 October 2025)
	[69]	Gold Atlas	Diffusion	https://github.com/QingLyu0828/diffusion_mri_to_ct_conversion (accessed on 20 October 2025)

* available on GitHub (https://github.com/, accessed on 20 October 2025) but not officially in the paper.

## 6. Evaluation Metrics

The performance of sCT generation is commonly assessed using three categories of metrics: intensity-based similarity, geometric fidelity, and dosimetry-based evaluation. Each of these captures complementary aspects of image quality and clinical usability, which are listed in Table 2.

### 6.1. Intensity-Based Metrics

To evaluate the voxel-wise intensity similarity between sCTs and reference CTs, the most used similarity metrics are Mean Absolute Error (MAE), Peak Signal to Noise Ratio (PSNR), and SSIM. Apart from these, Mean Error (ME) and Root Mean Square Error (RMSE) are commonly used to evaluate the similarity. These metrics directly evaluate differences in HU, with lower values indicating higher fidelity. Normalized Cross Correlation (NCC) is also reported in some literature to account for distributional differences in intensity.

### 6.2. Geometric-Based Metrics

Beyond voxel-level similarity, geometric fidelity is evaluated by comparing delineated anatomical structures between sCTs and reference CTs. The Dice Similarity Coefficient (DSC) quantifies volumetric overlap, while surface-based metrics, such as the Hausdorff Distance (HD) and Mean Absolute Surface Distance (MASD), measure boundary agreement. These metrics are especially relevant when sCTs are used for downstream tasks such as segmentation, where structural accuracy of OARs and target volumes is critical.

### 6.3. Dosimetry-Based Metrics

Since the goal of sCT generation is accurate radiotherapy planning, dose recalculation provides the most clinically relevant evaluation.

A commonly used metric is the dose difference (DD), which evaluates the difference between sCT-based and CT-based dose distributions. DD is typically computed either voxel-wise or as an average within specific ROIs. It can be reported as an absolute value (in Gy) or relative to a reference (in %), such as the prescribed or maximum dose. Some studies also report the dose pass rate, defined as the percentage of voxels where DD is below a specified threshold (e.g., 2% or 3%) [153].

Another widely used evaluation method is the dose–volume histogram (DVH), which plots the percentage of volume receiving at least a given dose. From the DVH, clinically relevant endpoints such as D_max_ (maximum dose), D_95%_ (dose covering 95% of the target volume), and V_20 Gy_ (volume percentage receiving ≥20 Gy) can be extracted and compared between sCT- and CT-based plans. These specific metrics are widely adopted in radiotherapy dose evaluation guidelines such as ICRU Report 83 [154] and QUANTEC (Quantitative Analyses of Normal Tissue Effects in the Clinic) [155], and have been used extensively in clinical sCT validation studies. Comparing DVH endpoints between sCT- and CT-based plans offers a practical way to assess dosimetric agreement in a clinically interpretable manner.

Gamma analysis is a widely adopted method that combines dose and spatial agreement into a single metric. It evaluates both the dose difference (in %) and the distance-to-agreement (in mm) simultaneously, reporting either the mean gamma index or the gamma pass rate (percentage of voxels with γ < 1). Gamma analysis can be performed in 2D or 3D, but its outcomes are highly dependent on parameters such as dose threshold, grid size, and voxel resolution, which complicates direct comparison across studies [156]. Most DL-based sCT images have reported average gamma pass rates (2%/2 mm) ranging from 92.0% to 99.5%. A gamma pass rate above 95% under these thresholds is typically considered acceptable [1,38].

## 7. Discussion

Deep learning has been applied for sCT generation in radiotherapy. Applications range from CBCT-based or MR-based workflows and the simulation-free scenario. Similarly, a variety of training strategies have been proposed to improve the performance, ranging from frequency-guided DL models to federated learning. This shows the trend of AI facilitating IGART. However, it is still challenging to apply AI algorithms in real-world clinical settings. In this section, we will discuss the challenges of clinical translation, along with data challenges, issues with current evaluation methods. Future directions such as benchmarking datasets and standardized pipelines will also be discussed.

### 7.1. Clinical Gap and Generalizability

Although good image fidelity and dose accuracy have been achieved by current DL models, the majority of the model development is performed under a specific narrow condition. They are usually confined to one anatomic location, treatment protocol, or homogeneous patient population. Different imaging scanners and data size also limit the generalizability. While few studies work with CycleGANs across anatomical sites [157,158,159,160], these are still largely constrained to site-specific protocols and cohorts.

Boily et al. recently analyzed a much larger dataset with 4000 patients and investigated model generalizability across age, sex and anatomical regions. They indicated that generalization is difficult and highly context-dependent [161]. Thus, we call for more coherent evaluation protocols designed for radiotherapy applications.

In addition, models such as GANs and Diffusion networks have high training costs while they obtain exceptional performance. Altalib et al. discussed that long runtimes and GPU demands make it hard to deploy these models in practice [14]. Clinical readiness requires a trade-off between performance and efficiency.

### 7.2. Data Challenges

High-quality paired datasets are essential for supervised sCT model training. However, in practice it is often difficult to obtain paired datasets. Due to the temporal gap between imaging sessions, there will be misalignment between two images. It is also not feasible to collect paired scans in typical clinical workflows [90,162]. These limitations have led to the growing interest in unpaired or even unsupervised approaches. Notable examples include CycleGANs [43], anatomy-regularized adversarial models [72] and latent-space Diffusion networks [94,95]. These models help improve training flexibility and reduce data dependency. However, their ability to preserve fine anatomical details and accurate HU values remains an open question [88].

Furthermore, as discussed in previous papers, data diversity is a long-standing problem that has not been well addressed [2,15]. Certain anatomical regions such as air pockets or dense bone are underrepresented in the training data, and this may lead to prominent errors when predicting sCT images. Federated learning has been suggested as a potential way to solve this issue [58,111,157], as it allows for broader population coverage without requiring data centralization.

### 7.3. Suitability of Evaluation Metrics

Most studies on sCT generation primarily reported intensity-based metrics such as MAE, PSNR, and SSIM. While these are useful for model benchmarking, they provide limited information on the clinical usage of DL models. For instance, small HU differences near OARs may have less impact on MAE but will heavily affect the dose distribution during treatment planning.

To address such spatially localized HU errors, gamma analysis has been widely adopted in many studies due to its joint evaluation of dose and spatial agreement. However, its clinical interpretability remains controversial due to dependency on arbitrary thresholds and limited correlation with clinical outcomes [156]. Hence, it is meaningful to report clinically interpretable metrics such as DVH deviation and HU accuracy in critical structures for clinical scenarios. However, such metrics are reported inconsistently across studies, and only a few studies related dosimetric errors to specific anatomical structures or model design choices [15]. To enable robust clinical validation, evaluation frameworks should be standardized to report not only intensity-based metrics but also clinically relevant ones. In the absence of a treatment plan, anatomy-based spatial descriptors such as the Overlap Volume Histogram (OVH) can be used as an alternative [163]. OVH captures the geometric relationship between targets and OARs without relying on a treatment plan and has shown potential in estimating achievable dose distributions [164].

### 7.4. Future Direction

To bridge the gap between experimental performance and clinical practice, future research can focus on the following directions:

- Open-Source Availability and Community Resources: Reproducibility remains a major challenge in deep learning-based sCT generation. Future research may prioritize the release of open-source codes and models, which would promote transparency and reproducibility. As highlighted in prior studies, the lack of code sharing hinders reproducibility in medical imaging AI and slows clinical translation compared to general computer vision, where open benchmarks and toolkits have driven rapid progress [165,166,167]. While some projects in Table 1 have released resources, efforts remain inconsistent. Community-maintained repositories and standardized pipelines are needed to support broader validation and adoption;
- Standardized Benchmarks: Large-scale and well-annotated benchmark datasets with consistent evaluation labels need to be established. Such datasets can be collected across multiple centers, imaging vendors, and treatment protocols to ensure fairness and generalizability [165];
- Multimodal Learning: By incorporating more image modalities, e.g., PET images, low field MRI, and CBCT images with different kV settings, various clinical scenarios can be covered. In this way, robustness of models can be improved and better OAR delineation on sCT can be achieved [77,124];
- Personalized medicine: To account for the diversity across different populations and institutions, approaches including federated learning can be further explored, which can enable the generation of high-quality, patient-tailored sCT [110,111];
- End-to-End Clinical Pipelines: As sCT synthesis is only one step in the radiotherapy workflow, it is critical to integrate it with downstream sections such as OAR delineation and treatment planning. A unified and end-to-end pipeline may improve reproducibility and facilitate smoother translation into clinical practice;
- Vendor Integration and Deployment: It is necessary to collaborate with treatment planning system vendors and hardware manufacturers to enable seamless integration of sCT synthesis into clinical workflows. Several vendor systems have already been proposed and evaluated, such as Syngo_BD (Siemens), MRI Planner (Spectronic), and MR-Box (Therapanacea) [168]. Models must be optimized for runtime efficiency, system compatibility, and real-time inference in clinical settings.

## Figures and Tables

**Table 2:** Formulas of evaluation metrics for synthetic image analysis.

Type of Metric	Metrics	Formula
Intensity-based	Mean Error	$ME=\frac{1}{n}\;\sum_{i=1}^{n}\left({sCT}_{i}-{CT}_{i}\right)$
	Mean Absolute Error	$MAE=\frac{1}{n}\;\sum_{i=1}^{n}\left|{sCT}_{i}-{CT}_{i}\right|$
	Mean Square Error	$MSE=\frac{1}{n}\;\sum_{i=1}^{n}({sCT}_{i}-{CT}_{i}{)}^{2}$
	Root Mean Square Error	$RMSE=\sqrt{\frac{1}{n}\;\sum_{i=1}^{n}({sCT}_{i}-{CT}_{i}{)}^{2}}$
	Peak Signal to Noise Ratio	$PSNR=10{log}_{10}\frac{{(2}^{b}-1{)}^{2}}{MSE}$
	Structural Similarity Index	$SSIM=\frac{\left(2\mu_{x}\mu_{y}+C_{1})(2\sigma_{xy}+C_{2}\right)}{\left(\mu_{x}^{2}+\mu_{y}^{2}+C_{1})(\sigma_{x}^{2}+\sigma_{y}^{2}+C_{2}\right)}$
	Normalized Cross Correlation	$NCC=\frac{1}{n}\sum \frac{\left(I_{sCT}-\mu_{sCT})(I_{CT}-\mu_{CT}\right)}{\sigma_{CT}\sigma_{sCT}}$
Geometric-based	Dice Similarity Coefficient	$DSC=\frac{2\;(V_{CT}\cap \;V_{sCT})}{V_{CT}+V_{sCT}}$
	Hausdorff Distance	$H\left(sCT,CT\right)=max\{\underset{x\in C_{\mathrm{CT}}}{\sup}\underset{y\in C_{\mathrm{sCT}}}{\inf}||x-y||,\;\underset{x\in C_{\mathrm{sCT}}}{\sup}\underset{y\in C_{\mathrm{CT}}}{\inf}\left||x-y||\right\}$
	Mean Absolute Surface Distance	$MASD\left(CT,sCT\right)=\frac{\left(\sum_{x\in C_{CT}}\min_{y\in C_{\mathrm{sCT}}}\left||x-y|\right|+\sum_{y\in C_{sCT}}\min_{x\in C_{\mathrm{CT}}}\left||y-x|\right|\right)}{\left|C_{CT}\right|+\left|C_{sCT}\right|}$

## Data Availability

Not applicable.

## Abbreviations

The following abbreviations are used in this manuscript:

CT	Computed Tomography
sCT	Synthetic Computed Tomography
MRI	Magnetic Resonance Imaging
CBCT	Cone-Beam Computed Tomography
OAR	Organ at risk
IGART	Image-guided adaptive radiotherapy
DL	Deep learning
CNN	Convolutional neural network
GAN	Generative adversarial network
FBCT	Fan-beam Computed Tomography
cGAN	Conditional generative adversarial network
GLFC	Global-Local Feature and Contrast learning
HU	Hounsfield unit
DVH	Dose-volume histogram
pCT	Planning Computed Tomography
DVF	Deformation vector field
MAE	Mean absolute error
PSNR	Peak signal to noise ratio
SSIM	Structural similarity index
